# Adult Day Service Use among Ethnic Minority Older Adults: An Updated Integrative Review

**DOI:** 10.1093/geroni/igab046.3458

**Published:** 2021-12-17

**Authors:** Yawen Li, Ling Xu, Jinyu Liu, Fei Sun

**Affiliations:** 1 California State University San Bernardino, San Bernardino, California, United States; 2 University of Texas at Arlington, Arlington, Texas, United States; 3 Columbia University, Columbia University, New York, United States; 4 Michigan State University, East Lansing, Michigan, United States

## Abstract

Adult day services (ADS) are a preferred care option for racial and ethnic minorities compared to other types of long-term care services in the United States. However, there is limited knowledge on minority ADS users. Focusing on minority older adults, this study aims to (a) identify facilitator and barriers of ADS use, and (b) examine ADS’s effect on health and wellbeing. Using Whittemore and Knafl’s methodology of integrative reviews, we searched relevant studies published between 2010 to 2020 in Ageline, PubMed, PsycINFO, CINAHL, Web of Science and Google Scholar and included 8 articles in this review after extensive screening and critical appraisal. Crowe Critical Appraisal Tool (CCAT) was used to assess methodological rigor of the studies included in this review. This review showed that individual factors of ADS use among minority older adult included functional impairment, diabetes, race, gender, and degree of loneliness. Organizational characteristics, such as availability of transportation services, bilingual nurses, peer support, and cultural activities, and structural factors including for-profit status and source of payment were also related to ADS use among minority older adults. Positive outcomes associated with ADS use were improved quality of life and sense of fulfillment. Better understanding of minority older adults’ experience with ADS will help tailor the services to better fit their cultural preferences and needs. Future research should move beyond individual-level factors to identify and address organizational and structural factors such as institutional structure, organization culture and practice impact on disparities and discrimination in services access and quality.

